# Acknowledgement to Reviewers of *Diseases *in 2017

**DOI:** 10.3390/diseases6010011

**Published:** 2018-01-22

**Authors:** Diseases Editorial Office

**Affiliations:** MDPI AG, St. Alban-Anlage 66, 4052 Basel, Switzerland

Peer review is an essential part in the publication process, ensuring that *Diseases* maintains high quality standards for its published papers. In 2017, a total of 35 papers were published in the journal. Thanks to the cooperation of our reviewers, the median time to first decision was 28 days and the median time to publication was 48 days. The editors would like to express their sincere gratitude to the following reviewers for their time and dedication in 2017:
Abenavoli, LudovicoMatsuura, BunzoAdriaenssens, EricMayhan, William G.Alves, SandraMcCoy, Sarah J. BreeseAmital, HowardMei, Ya-FangAthyros, VasiliosMestha, Lalit KAugust, Keith JMichelhaugh, Sharon KayBakonyi, TamasMiranda, KatrinaBalkema-Buschmann, AnneMonroy, FernandoBellezza, IlariaMorgante, GiuseppeBommakanti, GayathriNadiminty, NagalakshmiBortkiewicz, AlicjaNemeth, LynneBravo, LauraNolan, JohnBullon, PedroOlomu, IsokenBurger, MichaelOnyenwoke, Rob UCai, TommasoPan, MeixiaCatassi, CarloPang, Myung-GeolCeranowicz, PiotrPiche, Marie-EveChoy, Francis YMPistelli, FrancescoConigliaro, AlicePoluektova, LarisaConrad, ZachPulido, Olga MDas, AnupaRamos, SoniaDekker, MarloesRodenbeck, Stacey DineenEdwards, ErikaSabrina, NoelEverett, CarlosSaisho, YoshifumiFernandez, Maria LuzSenneville, ÉricGrant, WilliamSharland, MikeGray, AndrewSheng, XiuzhenGrimaldi, DomenicoShi, JiaqiGrinberg, DanielSkoien, RichardHarvey, AdamSteel, Laura F.Heggermont, WardStrohmaier, Walter L.Hioki, HirofumiSukocheva, OlgaIgaz, PeterTan, Bee KIslam, M. NurulTao-Hsin, TungJang, Byoung KukTeschke, RolfJansen, EugeneTiller, DanielJENA, PRASANTTso, For YueKaranis, PanagiotisUnchwaniwala, NuruddinKashiwagi, ShinichiroVan De Wetering, Marianne DesireeKim, Hyung SikVan Hoek, BartKlims-Zacas, DorothyVanamala, Jairam K.P.Kulus, MichałVilageliu, LLuisaKumar, KantaWahlestedt, ClaesKurdowska, Anna K.Wang, Cheng-pingKuwabara, MasanariWang, XinkunLamonaca, F.Ward, Peter ALang, ChimWegrzyn, GrzegorzLanghammer, BirgittaWeinreb, Neal J.Leibowitz, AvshalomWilusz, JeffLi, RuiXu, JingLi, WeijuanYang, ZhengyuLim, Jeong-AYiannakopoulou, EugeniaLin, Gen-MinYin, JunLonardo, AmedeoYoo, Tag KeunLouvet, AlexandreYoung, JeanineMachaczka, MaciejYumoto, HiromichiMakishima, MakotoZakaria, DalaMartini, MaurizioZavattari, PatriziaMartino, SabataZhong, XiaoboMarzullo, Paolo


